# Artificial intelligence for algorithmic trading digital assets: evidence from the Counter-Strike 2 skin market

**DOI:** 10.3389/frai.2025.1702924

**Published:** 2025-11-11

**Authors:** Federico Guede-Fernández, Yash Wagle, Pedro Dias, Ana Paula Giordano, Lúcio Henriques, Gonçalo Costa, Salomé Azevedo

**Affiliations:** 1 Value for Health CoLAB, Lisboa, Portugal; 2 Comprehensive Health Research Center, Faculdade de Ciências Médicas, Universidade NOVA de Lisboa, Lisboa, Portugal; 3 Laboratory for Instrumentation, Biomedical Engineering and Radiation Physics (LIBPhys), NOVA School of Science and Technology, Caparica, Portugal; 4 CATÓLICA-LISBON Research Unit in Business and Economics (CUBE), Lisboa, Portugal; 5 Exeedme, Braga, Portugal; 6 CEG-IST, Instituto Superior Técnico, Universidade de Lisboa, Lisboa, Portugal

**Keywords:** digital assets, deep learning, artificial intelligence, algorithmic trading, virtual economy, skins market, Counter-Strike 2

## Abstract

**Introduction:**

The Counter-Strike 2 skin market has developed into a multi-billion-dollar digital asset ecosystem, characterized by high volatility, low liquidity, and pricing inefficiencies that differ substantially from traditional financial markets. Despite the growing economic relevance of virtual items, no previous study has systematically examined the use of artificial intelligence for skin trading.

**Methods:**

This work designs and evaluates an automated trading system that applies deep learning models, specifically Long Short-Term Memory networks and Neural Hierarchical Interpolation for Time Series, to forecast skin prices and guide trading decisions. A dataset of 12,000 unique skins from the Steam Market, covering the period from May 2024 to April 2025, was collected using the CSGOskins.gg application programming interface. To reflect real market conditions, the trading strategy incorporated the Steam Market restrictions of a seven-day minimum holding period and a ten percent transaction cost, and was benchmarked against a traditional buy-and-hold strategy. Backtesting was performed multiple time horizons of two, three, and 6 months. Portfolio selection was based on risk and return criteria, including a Sharpe ratio greater than one, a Sortino ratio greater than two, and a return on investment above five percent.

**Results:**

Artificial intelligence consistently outperforms buy-and-hold, particularly in smaller, more concentrated portfolios and over longer time horizons. For example, in 6-month simulations, artificial intelligence portfolios achieved returns approaching 20%, compared to 5% to 10% for buy-and-hold, with excess returns as high as 75% in small portfolios. Larger portfolios reduced absolute returns but improved risk-adjusted performance, confirming that diversification enhances stability while diluting raw profitability. Analysis of portfolio composition by rarity further revealed that artificial intelligence favors moderately rare and liquid skins such as Mil-Spec, resembling mid-cap equity investment strategies, while buy-and-hold accumulates rarer skins, analogous to small-cap holdings that rely on scarcity premiums.

**Discussion:**

These findings highlight that even in virtual goods markets, the trade-offs between return, risk, and diversification reflect established principles of modern portfolio theory. The study demonstrates both the feasibility and the potential of artificial intelligence-based trading systems in the Counter-Strike 2 skin economy, contributing methodological advances and practical insights for participants in this emerging digital asset market.

## 1 Introduction

The Counter-Strike 2 (CS2) skin market has emerged as a sophisticated digital asset exchange, presenting opportunities for algorithmic trading systems. Unlike traditional financial markets, which rely on centralized exchanges and order books, CS2 skins are traded through peer-to-peer transactions. This decentralized structure creates unique inefficiencies that can be exploited through artificial intelligence (AI)-driven trading strategies. A growing body of research has focused on applying artificial intelligence to financial forecasting, emphasizing the potential for deep learning models to enhance price prediction accuracy and optimize trading strategies (Chopra and Sharma, 2021).

The CS2 skin market, with an estimated valuation of $4.1 billion as of February 2025, operates within a framework that blends elements of collectibles markets and financial derivatives (D'Anastasio, 2025). Unlike traditional exchanges, where pricing mechanisms are driven by centralized order books, the skin market relies on direct peer-to-peer transactions, leading to significant price discrepancies across different platforms (Thorhauge and Nielsen, 2021). These inefficiencies create opportunities for algorithmic trading strategies capable of exploiting short-term arbitrage opportunities.

The valuation of CS2 skins is influenced by multiple factors, with artificial scarcity playing a dominant role (Ladeira et al., 2023). In addition to scarcity, cosmetic appeal introduces a subjective dimension to pricing, as buyer preferences for particular patterns and wear conditions can create price variations for identical skin types. External influences further contribute to price fluctuations, particularly the impact of e-sports events and developer updates (Jo and Lewis, 2024). Championship victories often drive demand for related skins, while game updates introduce exogenous shocks that significantly alter market valuations. These dynamics distinguish skin markets from traditional financial securities, necessitating specialized predictive models.

In recent years, artificial intelligence has revolutionized algorithmic trading in traditional financial markets. Techniques such as recurrent neural networks (RNNs), long short-term memory (LSTM) networks, and more recently, advanced architectures like Neural Hierarchical Interpolation for Time Series (NHITS), have demonstrated remarkable success in modeling complex temporal patterns and forecasting asset prices (Hochreiter and Schmidhuber, 1997; Challu et al., 2023). These AI-based approaches enable the development of automated trading systems that can adapt to rapidly changing market conditions, optimize portfolio allocations, and manage risk more effectively than traditional rule-based strategies (Siami-Namini et al., 2018; Schäfer et al., 2025).

Despite the proven efficacy of AI-based trading algorithms in equities, commodities, and cryptocurrencies (Amirzadeh et al., 2022; Ozer and Sakar, 2022), their application to digital gaming assets such as CS2 skins remains largely unexplored. The unique characteristics of the skin market, including high volatility, illiquidity, and the influence of non-financial factors, present both challenges and opportunities for the deployment of AI-based trading systems. Recent studies in adjacent fields, such as non-fungible tokens (NFTs) and virtual item markets, suggest that machine learning can be leveraged to identify pricing inefficiencies, forecast demand surges, and automate trading decisions with significant success (Xiong et al., 2024; Horky et al., 2022; Dawod et al., 2023).

The goal of this work is to design, develop, and validate an automated trading system for CS2 skin trading, tailored to maximize capital gains. The proposed system leverages data-driven trading strategies, ensuring that buy and sell decisions align with market trends and predefined risk levels. By incorporating financial performance metrics and risk-adjusted evaluation criteria, the bot aims to identify and execute profitable trades with optimal timing. The system is designed to dynamically adjust its strategy based on market conditions, allowing for efficient portfolio management and risk mitigation. Ultimately, this approach enhances the profitability and reliability of skin trading for users by providing a structured, algorithmic method to navigate the CS2 market.

The remainder of this paper is organized as follows: Section 2 presents related work in the field, Section 3 details the methodology for data collection and the development of AI-based trading strategies. Section 4 presents the results in terms of investment profitability, risk, time horizons, and portfolio construction. This section also discusses these findings in the context of existing literature and market dynamics, and Section 5 concludes with the main contributions of this work.

## 2 Related work

Algorithmic trading strategies, such as statistical arbitrage and pairs trading, have been widely adopted in traditional financial markets to exploit pricing inefficiencies between correlated assets (Thomaidis et al., 2006). These methods typically depend on the ability to take both long and short positions, allowing traders to profit from price divergences and subsequent convergences (Thomaidis et al., 2006). However, such approaches are not directly transferable to the CS2 skin market due to structural constraints. Specifically, the absence of short-selling mechanisms for skins eliminates the possibility of hedging through negative positions. Additionally, the Steam marketplace enforces a seven-day trading restriction, which precludes high-frequency trading strategies that rely on rapid execution and order turnover (Thorhauge, 2023). These limitations necessitate the development of alternative, AI-driven trading strategies that are specifically adapted to the unique characteristics of virtual goods markets.

Artificial intelligence, particularly deep learning, has significantly advanced the field of financial time series forecasting (Lin and Marques, 2024). Models such as RNN and LSTM have demonstrated strong capabilities in capturing non-linear temporal dependencies in asset prices. Ensemble methods, which combine multiple machine learning models, have further improved prediction robustness and mitigated overfitting by leveraging diverse perspectives on historical data (Song et al., 2024). While these AI methodologies have been extensively applied to equity and cryptocurrency markets, their application to virtual asset markets such as CS2 skins, remains largely unexplored. Recent studies in gaming economies suggest that AI can be effective for dynamic pricing, trend prediction, and fraud detection, but there is a notable absence of research focused on predictive modeling and automated trading for CS2 skins specifically.

Modern AI trading bots in the crypto and digital asset space are designed to optimize not just for absolute returns but for risk-adjusted performance, with the Sharpe ratio serving as a primary benchmark. These bots use machine learning to dynamically adjust position sizes, stop-losses, and asset allocations in response to changing volatility and market regimes, directly targeting higher Sharpe ratios and lower drawdowns. Systematic reviews confirm that AI trading systems are routinely backtested and evaluated using risk-adjusted metrics, including the Sharpe ratio, Sortino ratio, and maximum drawdown, to ensure robust performance across diverse market conditions (Dakalbab et al., 2024).

Effective trading systems must balance profitability with risk management. In traditional finance, the Sharpe ratio serves as a standard metric for evaluating risk-adjusted returns. However, the CS2 skin market presents unique challenges: the value of skins is closely linked to the game's lifecycle, player demand, and periodic game updates, necessitating specialized, time-weighted risk models. Portfolio diversification strategies must consider not only price correlations between different skins but also their sensitivity to developer interventions and shifts in competitive gameplay meta. Constructing a resilient portfolio thus requires a nuanced approach, balancing high-value, rare items with more liquid, mid-tier assets to mitigate exposure to market volatility and unpredictable demand shocks (Reichenbach, 2025).

The use of artificial intelligence and machine learning in studying virtual economies has gained increasing attention as these markets evolve into complex, data-rich ecosystems that mirror real-world economic structures. Virtual economies in online games operate with mechanisms analogous to traditional markets, emphasizing the need for quantitative and predictive modeling approaches (Chambers, 2011). More recent studies have applied AI to measure inflation, price volatility, and transaction dynamics within digital marketplaces. A deep learning framework was proposed to assess inflation and predict pricing behavior in virtual economies (Stephens and Exton, 2021). Complementary studies have examined how AI contributes to financial insights in metaverse and in-game economies, in-game product pricing was modeled to identify optimal valuation strategies, offering a basis for integrating AI-based price forecasting in virtual markets (Budak and Özen, 2022). The behavioral dimension has also been addressed through machine learning analyses of user trading patterns (Aspembitova et al., 2021), which modeled strategy clusters in cryptocurrency markets, which is an approach equally relevant to understanding behavioral heterogeneity in virtual asset trading. These studies provide empirical support for applying AI to the CS2 skin market, where complex interactions among rarity, player behavior, and liquidity drive asset valuation.

Despite the growing popularity and economic significance of virtual item trading, there is currently no published research investigating the use of AI-based trading strategies for CS2 skins. Existing work has focused on automating transactions and ensuring fair pricing through trading bots, but comprehensive studies on predictive modeling, automated decision-making, and portfolio optimization in this domain are lacking. This gap stresses the need for research that adapts and extends AI methodologies to address the specific challenges and opportunities of the CS2 skin market.

## 3 Methods

### 3.1 Data collection

**Figure 1:**
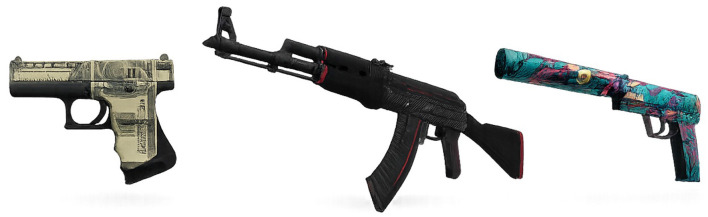
Representative examples of CS2 skins across rarity levels, arranged from common to ultra-rare. From left to right: (1) Glock-18 — Franklin (Well-Worn) (Industrial Grade), (2) AK-47 — Redline (Field-Tested) (Mil-Spec), and (3) StatTrak^TM^ USP-S — Monster Mashup (Well-Worn) (Restricted). All images are^©^ Valve Corporation, retrieved from the public Steam Community Market for non-commercial, academic illustration purposes only.

Historical price data for CS2 skins were collected from the Steam Market via the CSGOskins.gg API, which provides daily price information for individual items. Due to API limitations, the dataset covers the period from May 1, 2024, to April 30, 2025, comprising 12,000 unique skins. Only Steam Market listings were considered to ensure price consistency. The dataset was partitioned into training (8.3%), validation (41.6%), and test (50%) sets to capture diverse market conditions, optimize model parameters, and rigorously evaluate out-of-sample performance. This split mitigates overfitting and supports reliable assessment of the trading strategies. This split configuration was selected to ensure a sufficient horizon for hyperparameter optimization and robust out-of-sample testing, given the limited 1year historical dataset available (May 2024–April 2025). Figure 1 provides representative visual examples of CS2 weapon skins, illustrating the spectrum of rarity levels and associated aesthetic features.

To ensure data integrity, only skins with complete daily price records for the full observation period (May 2024 to April 2025) were included in the analysis. No imputation, interpolation, or normalization procedures were applied, as each forecasting model was trained independently for each skin using its raw daily price data. This approach avoided potential biases introduced by synthetic data filling or scaling transformations, allowing the models to learn directly from the natural market variability. Outliers were retained, as they represent genuine fluctuations in market behavior rather than data errors.

### 3.2 Artificial intelligence based forecasting models: NHITS and LSTM

To generate trading signals, we implemented two state-of-the-art deep learning models: NHITS and LSTM networks. Both models were independently trained and validated for each individual skin item, allowing the forecasting process to capture the unique price dynamics, volatility, and trading patterns associated with each asset. NHITS is designed to capture long-term dependencies and seasonality in time series data, and has demonstrated superior performance on various forecasting benchmarks (Challu et al., 2023). In our study, NHITS was trained on historical price spreads to produce next-day forecasts. LSTM networks are well-established for modeling sequential data and short-term trends in financial time series (Hochreiter and Schmidhuber, 1997; Siami-Namini et al., 2018). The LSTM model was used to predict short-term price movements and spread changes. The use of the LSTM and NHITS models was guided by the objective of assessing the feasibility of AI-based forecasting in the CS2 skin market using two distinct deep learning paradigms. LSTM represents a recurrent neural network architecture particularly suited for capturing sequential dependencies and temporal dynamics, while NHITS is a multilayer perceptron-based hierarchical model designed for efficient and interpretable long-term forecasting. The purpose was not to identify the optimal forecasting framework but to establish a methodological baseline for subsequent exploration. For both models, hyperparameter optimization was conducted using Bayesian optimization (Snoek et al., 2012). To ensure computational efficiency and feasibility for real-time trading, only five distinct hyperparameter configurations were sampled and evaluated for each model and each skin. The specific parameter ranges are detailed in Table 1. Despite the individualized training, training and inference for each model and each skin were completed in under 1 min.

**Table 1:** Hyperparameters for NHITS and LSTM models.

**Model**	**Parameters**	**Description**	**Values**
NHITS	input_size	Length of input window	30 (6M), 15 (3M), 10 (2M)
	start_padding_enabled	Whether to enable start padding	True
	n_blocks	Number of blocks in the stack	5 × [1]
	mlp_units	Number of units in each MLP layer	5 × [[64, 64]]
	n_pool_kernel_size	MaxPooling kernel size	[1,1,1,1,1], [2,…], [4,…], [8,4,2,1,1]
	n_freq_downsample	Interpolation expressivity ratios	[8,4,2,1,1], [1,1,1,1,1]
	learning_rate	Initial learning rate (log-uniform distribution)	loguniform(1e-4, 1e-2)
	scaler_type	Scaler type used	None
	max_steps	Max number of training iterations	500
	batch_size	Number of series in batch	[1, 4, 10]
	windows_batch_size	Number of windows in batch	[128, 256, 512]
	random_seed	Random seed	randint(1, 20)
LSTM	input_size	Length of input window	30 (6M), 15 (3M), 10 (2M)
	encoder_hidden_size	Hidden size of LSTM cells	[64, 128]
	encoder_n_layers	Number of LSTM layers	[2, 4]
	learning_rate	Initial learning rate (log-uniform distribution)	loguniform(1e-4, 1e-2)
	scaler_type	Scaler type used	robust
	max_steps	Max number of training iterations	500
	batch_size	Number of series in batch	[1, 4]
	random_seed	Random seed	randint(1, 20)

For each skin, the model with the lowest validation mean quantile loss (MQLoss) was selected for trading. MQLoss is a regression loss function commonly used in time series forecasting to evaluate the accuracy of predicted quantiles. Unlike traditional metrics such as mean squared error or mean absolute error, which assess the average magnitude of prediction errors, MQLoss specifically measures how well the model predicts a particular quantile (the median or the 90th percentile) of the target distribution. This is particularly useful in financial forecasting, where understanding the distribution of possible outcomes—rather than just the average—is crucial for risk management and decision-making (Matheson and Winkler, 1976; Koenker and Bassett, 1978). Mathematically, the quantile loss for a quantile q is defined as


$$\text{Quantile Loss}=\frac{1}{N}\sum_{i=1}^{N}\max(q\cdot (y_{i}-{\hat{y}}_{i}),(q-1)\cdot (y_{i}-{\hat{y}}_{i}))$$ (1)

where *y*_*i*_ is the true value, ŷ is the predicted value, and *N* is the number of observations. By minimizing the MQLoss for median level on the validation set for each skin, the selected model is optimized not just for average accuracy, but for robust performance across different possible market scenarios.

Each skin was modeled and trained independently using two forecasting architectures. For each skin, both models were trained and validated separately, and the model achieving the highest return on investment (ROI) during the validation phase was selected for subsequent out-of-sample performance evaluation.

### 3.3 Automatic trading strategy design

The trading strategies were implemented and backtested using the Backtrader Python framework. To comply with Steam Market rules, a seven-day minimum holding period was enforced: trades could only be executed at least seven days after the previous transaction. The strategy utilizes next-day price forecasts to inform entry and exit decisions, aiming to maximize returns while minimizing transaction frequency and costs.

#### 3.3.1 Trading logic

At each time step *t*, the model retrieves the actual closing price and the predicted price for *t*+7. If no position is open and the predicted price exceeds the current price (after accounting for transaction costs), a buy order is triggered. If a position is open and the predicted price is lower than the current price, a sell order is executed. Portfolio value is updated after each trade to track cumulative performance.

#### 3.3.2 Holding period and transaction cost

A strict seven-day holding period is enforced, reflecting Steam's trading restrictions. Trades cannot be executed more frequently than once every seven days per asset. A transaction selling cost of 10% per trade is incorporated to simulate real-world Steam conditions and assess strategy robustness. The buy-and-hold strategy, where an asset is purchased at the start of the period and held throughout, serves as the baseline for comparison.

### 3.4 Benchmarking and performance metrics

To rigorously evaluate the effectiveness of the AI-based trading strategies, performance was bench-marked against a buy-and-hold baseline using a comprehensive set of return and risk-adjusted metrics. These metrics provide a multi-faceted view of profitability, risk exposure, and strategy robustness. Return on Investment (ROI) measures the percentage gain or loss relative to the initial capital invested. It is a straightforward indicator of profitability, calculated as:


$$\begin{array}{l} \text{ROI}=\frac{\text{Net Profit}}{\text{Initial Investment}}\times 100 \end{array}$$ (2)

While ROI indicates absolute returns, it does not account for the risk or volatility taken to achieve those returns, which is critical in volatile markets such as virtual assets. Sharpe ratio evaluates risk-adjusted returns by comparing the excess return of the strategy over a risk-free rate to the standard deviation (volatility) of returns. It is defined as:


$$\begin{array}{l} \text{Sharpe Ratio}=\frac{E\left[R_{p}-R_{f}\right]}{\sigma_{p}} \end{array}$$ (3)

where *R*_*p*_ is the portfolio return, *R*_*f*_ the risk-free rate, and σ_*p*_, the standard deviation of portfolio returns. A higher Sharpe ratio indicates more efficient compensation for risk, with values above 1 generally considered good (Schäfer et al., 2025; Sharpe, 1966). This metric is essential for assessing whether the AI models improve returns without disproportionately increasing risk. The Sortino ratio refines the Sharpe ratio by focusing solely on downside volatility, negative deviations from a target return, rather than total volatility. It is computed as:


$$\begin{array}{l} \text{Sortino Ratio}=\frac{E\left[R_{p}-R_{f}\right]}{\sigma_{d}} \end{array}$$ (4)

where σ_*d*_ is the standard deviation of downside. This provides a more accurate measure of downside risk, which is particularly relevant for trading strategies sensitive to drawdowns.

In addition, backtesting was conducted over multiple time windows (last two, three, and 6 months) to ensure robustness across market regimes. For each window, the above metrics were computed on the subsequent out-of-sample period to simulate realistic trading scenarios, avoid lookahead bias and simulate realistic trading conditions. Performance metrics were computed over the out-of-sample evaluation period following model training and validation. For the 6-month analysis, this corresponds to a 1-month training window, a 5-month validation period used for model selection, and a subsequent 6-month testing window where trading decisions were executed. The same relative proportions were maintained for the shorter two- and 3-month configurations, with metrics calculated over their respective testing horizons. This design ensured that all reported results reflect true out-of-sample performance, independent of model fitting or hyperparameter tuning phases.

### 3.5 Portfolio construction

Portfolios were constructed by selecting skins based on historical performance. Optimization criteria included maximizing the Sharpe ratio, ROI, and Sortino ratio, with thresholds set to ensure both profitability and risk control (Sharpe ratio ≥ 1, Sortino ratio ≥ 2, ROI ≥ 5%). Portfolios consisted of one unit per selected skin, and profitability was assessed over multiple time horizons (2 months, 3 months, and 6 Months). The impact of skin rarity on portfolio performance was also analyzed. The thresholds were selected for portfolio inclusion following the criteria: Sharpe ratio ≥ 1, Sortino ratio ≥ 2, and ROI ≥ 5%. These thresholds were applied in the portfolio construction process to prioritize not only profitability but also the efficiency and quality of returns, in line with best practices in quantitative finance and risk management. The detailed description for each criteria is next discussed:

- Sharpe Ratio ≥ 1: Sharpe Ratio measures the excess return per unit of total portfolio volatility, providing a standardized means to compare risk-adjusted performance across different strategies. A Sharpe ratio above 1 is widely regarded as indicative of a good risk-adjusted return, meaning the portfolio delivers returns that sufficiently compensate for the risk taken. Portfolios with a Sharpe ratio below 1 may not be offering adequate reward for the volatility endured, while those above 1 are generally considered attractive to investors seeking efficient risk management.
- Sortino Ratio ≥ 2: Sortino Ratio refines the Sharpe ratio by focusing exclusively on downside risk (the standard deviation of negative returns) rather than total volatility. This makes it particularly relevant for strategies or assets with asymmetric return distributions or frequent positive price spikes, as is common in speculative or high-growth markets. A Sortino ratio above 2 is typically interpreted as a sign of strong risk-adjusted performance, indicating that the portfolio generates high returns relative to its downside risk exposure.
- ROI ≥5%: ROI is a basic measure of absolute profitability. Setting a minimum threshold of 5% ensures that only portfolios with meaningful net gains, after accounting for transaction costs and market frictions, are considered. This filter helps exclude portfolios that may perform well on a risk-adjusted basis but fail to deliver sufficient absolute returns to justify active trading.

Both the AI-based and BH portfolios were constructed from the same initial pool of candidate skins but differ in the selection mechanism used to form the final holdings. Skins were first filtered using performance-based inclusion criteria (Sharpe ratio ≥ 1, Sortino ratio ≥ 2, ROI ≥ 5%) to ensure that only statistically robust and actively traded items were considered. After filtering, all eligible skins were ranked by their ROI, providing a consistent and quantitative basis for selection. The AI strategy dynamically selected skins from this ranked list based on predicted short-term profitability, whereas the BH portfolio included the top-ranked skins without predictive adjustments. Consequently, the two portfolios may contain partially overlapping but not identical compositions, reflecting their distinct investment philosophies. This approach enables a fair yet realistic comparison between an active, prediction-driven strategy and a passive benchmark that mirrors conventional market exposure.

### 3.6 Rarity

CS2 skin rarity, which directly influences market value, was recorded for each asset in the portfolio. The CS2 rarity system includes eight tiers, from Consumer Grade (most common) to Extraordinary (rarest), as detailed in Table 2. Rarity not only drives the price premium of individual skins but also exerts a significant influence on market liquidity. Assets in the highest rarity tiers often experience elevated price levels and increased volatility, as their scarcity creates conditions of thin market depth and wider bid-ask spreads. This reduced depth of order books limits trade frequency, making rare skins relatively illiquid despite their high notional values. In contrast, lower-rarity items function as more liquid instruments, with higher trading volumes, narrower spreads, and greater ease of convertibility into real or platform-specific currency (Chordia et al., 2000; Mago et al., 2017). Therefore, the skins rarity was analyzed to assessment of how rarity affects both individual skin performance and overall portfolio outcomes.

**Table 2:** Classification of CS2 skin rarities and their characteristics.

**Type of rarity**	**Description**
Consumer Grade	The most common skins, typically received as random drops after matches or leveling up. They feature minimal design changes and have low market value.
Industrial grade	Slightly less common than Consumer Grade, with modest design enhancements. Also obtained through post-match drops and usually inexpensive.
Mil-Spec	Also called “Rare” skins, often found in weapon cases. They have more intricate designs and serve as the baseline for many skin collections.
Restricted	Less common than Mil-Spec, featuring more elaborate designs. Typically obtained from weapon cases and have moderate market value.
Classified	A higher rarity tier with infrequent drops from weapon cases. Known for unique and visually appealing designs that are more desirable.
Covert	Among the rarest in standard weapon cases. Coveted for their distinctive, elaborate designs and high market value.
Contraband	Skins removed from the game and no longer obtainable through normal means. Their rarity and exclusivity increase their market value.
Extraordinary	Includes ultra-rare items like knives and gloves. Extremely rare and highly valued within the CS2 community.

## 4 Results and discussion

### 4.1 Performance evaluation of AI-based and buy-and-hold trading strategies

**Figure 2:**
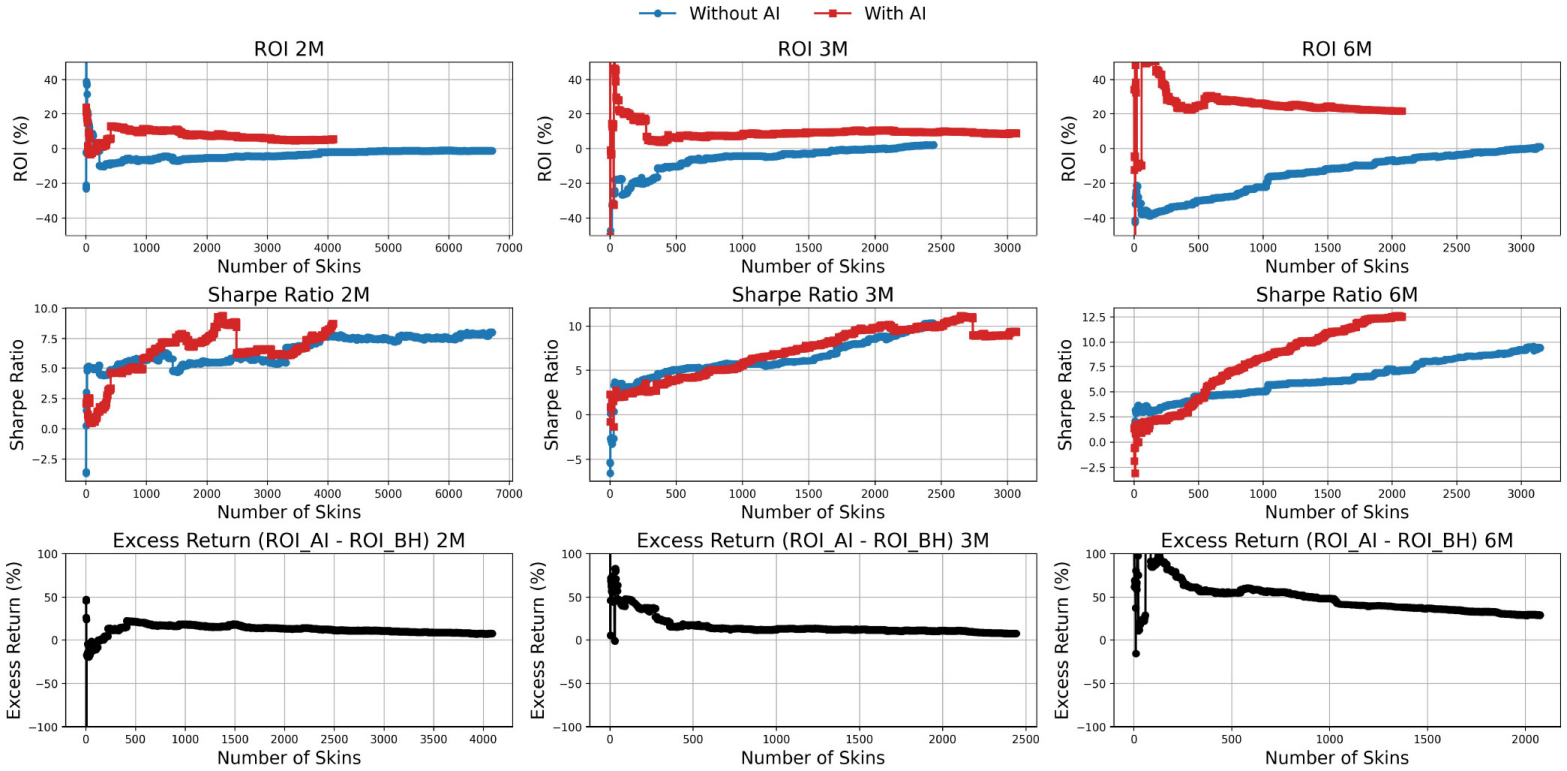
Comparison of portfolio performance between the artificial intelligence (AI)-driven strategy (red) and the buy-and-hold benchmark (blue) over 2-, 3-, and 6-month horizons. The **top, middle**, and **bottom rows** show return on investment (ROI), Sharpe ratio, and excess return, respectively, across increasing portfolio sizes.

Figure 2 presents the ROI, Sharpe ratio, and excess return for various portfolio strategies based on CS2 skin trading. The analysis was conducted under a standardized trading configuration: each skin was held for seven days between purchase and sale, with only one unit of each skin acquired, and a ten percent commission applied at the point of sale. Skins were selected according to predefined criteria (Sharpe ratio greater than one, Sortino ratio greater than two, and ROI above five percent) to ensure that strong candidates were included in each portfolio. Performance was evaluated across three distinct investment horizons: 2 months (2M), 3 months (3M), and 6 months (6M), using historical data available up to April 30, 2025.

The figure compares the performance of the AI-based trading strategy with a BH benchmark across different portfolio sizes and time horizons. Several consistent patterns emerge. Increasing the number of skins in the portfolio is associated with a decline in ROI for both strategies. For example, in the 2-month horizon, the AI strategy achieves ROI values around 15–20% in smaller portfolios before stabilizing close to 10% as portfolio size exceeds 3,000 skins. By contrast, the buy-and-hold strategy hovers near zero in small portfolios and approaches approximately 5% in larger ones. Importantly, ROI for the AI method increases with longer horizons, reaching values above 20% in the 6-month evaluation, while BH remains comparatively flat around 5–10%. This confirms that AI strategies consistently outperform buy-and-hold in terms of profitability, with the advantage becoming more pronounced over extended horizons, although diversification reduces its magnitude as portfolios expand (Sun and Govind, 2017).

Sharpe ratios improve with portfolio size for both approaches, reflecting enhanced risk-adjusted performance through diversification. In the 2-month horizon, AI-driven portfolios reach Sharpe ratios around 7-8, while buy-and-hold portfolios stabilize near similar levels. At three and 6 months, however, AI portfolios exhibit a clear advantage, with Sharpe ratios rising above 10–12 in large portfolios, compared with 7–9 for buy-and-hold. This demonstrates that AI strategies deliver superior risk-adjusted returns, particularly over longer horizons, consistent with findings in traditional financial markets where diversification reduces idiosyncratic risk while improving stability.

The excess return (*ROI*_*AI*_−*ROI*_*BH*_) further illustrates the performance gap. At the 2-month horizon, excess returns fluctuate widely in small portfolios, occasionally exceeding 50%, before stabilizing around 5–10% for portfolios larger than 2,000 skins. With longer horizons, the advantage of AI compounds: in the 6-month evaluation, small AI-driven portfolios achieve excess returns close to 75%, while larger, diversified portfolios still maintain an advantage of roughly 10–15%. These patterns align with evidence from equity markets, where predictive or active trading strategies tend to generate higher abnormal returns in concentrated portfolios, but diversification moderates these benefits as portfolio size grows (Sutiene et al., 2024; Chen, 2025).

### 4.2 Comparative analysis of portfolio composition by skin rarity

**Figure 3:**
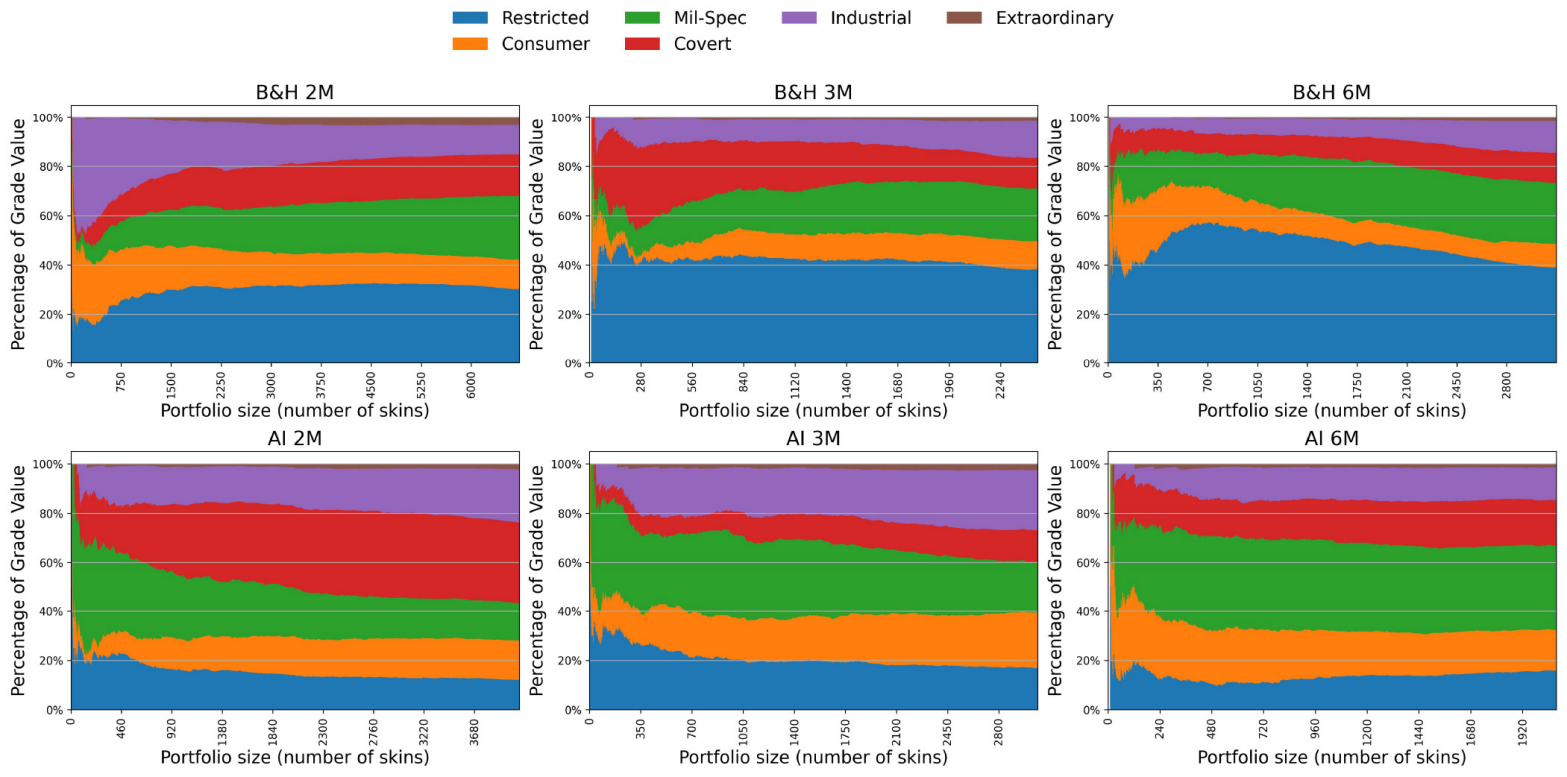
Portfolio composition by skin rarity in the Counter-Strike 2 market under buy-and-hold **(top row)** and AI-driven **(bottom row)** strategies, across 2-, 3-, and 6-month horizons. Each stacked area plot shows the relative share of Consumer, Industrial, Mil-Spec, Restricted, Covert, and Extraordinary skins as portfolio size increases.

Figure 3 illustrates the evolution of portfolio composition across different rarity categories in CS2, comparing the Buy-and-Hold benchmark with the AI-driven trading strategy over horizons of 2, 3, and 6 months. Rarity in CS2 is structured in an ascending hierarchy from more common to more exclusive items: Consumer Grade, Industrial Grade, Mil-Spec, Restricted, Covert, and Extraordinary.

Across both methods (AI and BH) and all time horizons, the portfolios exhibit a layered composition reflecting this rarity structure. In smaller portfolios, common items such as Consumer and Industrial skins dominate, but as portfolio size increases, the share of more valuable categories, particularly Restricted, Mil-Spec, and Covert, grows in importance. This trend stabilizes for larger portfolios, where the rarity distribution converges toward relatively stable patterns, especially visible at the 3- and 6-month horizons. This suggests that diversification drives portfolios toward a composition that approximates the broader market structure.

When comparing strategies, clear differences emerge. AI-driven portfolios exhibit a strong and consistent tilt toward Mil-Spec and Consumer skins. For example, in the 2-month horizon, Mil-Spec accounts for roughly 35–45% of total portfolio value in AI, compared with around 20–25% in Buy-and-Hold. This indicates that the AI strategy systematically favors moderately rare and liquid items, likely reflecting their balance between affordability, tradability, and predictive profitability. By contrast, Buy-and-Hold portfolios place greater weight on higher-rarity items such as Restricted and Covert, which together represent about 50–60% of value in the 3-month and 6-month horizons, compared with 30–40% in the equivalent AI cases.

A particularly notable divergence appears in the 6-month AI horizon. Here, Consumer and Mil-Spec together exceed 60% of the total portfolio value, while Restricted and Covert fall below 30%. This reallocation toward more common categories suggests that, over longer horizons, the AI strategy prioritizes liquidity and turnover rather than exposure to scarcity-driven appreciation. In contrast, the Buy-and-Hold approach continues to accumulate rarer skins, consistent with its passive reliance on scarcity premiums.

This divergence between AI and Buy-and-Hold parallels well-established phenomena in financial markets. The AI strategy's preference for Mil-Spec and Consumer skins resembles institutional investors' bias toward mid-cap and liquid equities, which combine reasonable return potential with market depth. Conversely, Buy-and-Hold's tilt toward Restricted and Covert parallels small-cap and illiquid equity exposure, where scarcity and liquidity risks drive long-term premiums. Prior financial studies support this interpretation: liquidity risk and size effects are recognized as key drivers of returns, with illiquid assets demanding higher expected returns (Choy and Wei, 2020), while more liquid mid-cap assets often deliver superior risk-adjusted outcomes over certain horizons (Pástor and Stambaugh, 2003). Thus, the AI's systematic allocation toward less rare categories can be viewed as a rational preference for liquidity and turnover, whereas Buy-and-Hold emphasizes scarcity-value accumulation.

### 4.3 Positioning AI-based trading within financial and digital markets

The findings of this study position CS2 skin trading within the broader landscape of financial asset management, revealing that market behaviors and portfolio dynamics in virtual economies closely follow principles established in modern finance. The AI-based strategy's systematic preference for moderately rare and liquid items, such as Mil-Spec skins, parallels mid-cap equity investment strategies, which optimize the balance between liquidity and expected returns. Conversely, the buy-and-hold strategy's concentration in rarer, less liquid skins mirrors small-cap investing, where scarcity premiums and illiquidity underpin long-term value appreciation. The consistent application of risk-adjusted performance metrics—such as the Sharpe and Sortino ratios—further reinforces these analogies, situating digital asset trading firmly within the framework of portfolio theory and emphasizing that the trade-offs between risk, return, and diversification extend beyond traditional financial markets.

These insights also align with developments in cryptocurrency and NFT markets, AI has been successfully deployed for price forecasting, volatility modeling, and automated trading (Nelson et al., 2017; Fischer and Krauss, 2018; Aydoğdu and Aydin, 2024). Similar to CS2 skins, both cryptocurrencies and NFTs exhibit high volatility, limited liquidity, and value driven by scarcity and aesthetic appeal. The use of deep learning architectures such as LSTM and NHITS in this study reflects a shared methodological foundation with AI models in decentralized finance, highlighting the adaptability of these techniques to alternative and behaviorally influenced digital markets.

From a practical perspective, the results demonstrate the potential of AI-driven trading frameworks to extend beyond conventional securities toward virtual and hybrid digital assets. The combination of backtesting, quantitative risk thresholds, and portfolio optimization techniques used here provides a replicable foundation for FinTech innovation. These systems can support automated asset allocation, cross-market arbitrage, and risk-adjusted portfolio construction-tools that are increasingly relevant for asset managers navigating emerging digital economies. As the boundaries between gaming, blockchain, and finance continue to blur, AI-enhanced trading strategies offer new opportunities for portfolio diversification, liquidity management, and sustainable growth within the FinTech ecosystem.

The CS2 skin market represents a niche, player-driven digital economy with structural properties distinct from conventional financial markets. Trading activity is largely governed by game engagement cycles, cosmetic preferences, and limited supply mechanisms, resulting in lower liquidity and greater price sensitivity to non-financial factors such as esports events or game updates (Lehdonvirta, 2009; Hamari and Keronen, 2017). These behavioral and scarcity-driven dynamics explain the AI strategy's preference for moderately rare and more liquid skins, which provide a more stable basis for short-term predictive modeling compared to highly rare items, whose prices are dominated by collector behavior and infrequent transactions. Similar findings have been reported in studies of virtual goods and NFT markets, where AI and econometric models perform better on assets with higher trading frequency and clearer price signals (Nadini et al., 2021; Dowling, 2022).

### 4.4 Limitations and future work

While these findings reinforce the feasibility of AI-driven trading in digital goods markets, several limitations must be acknowledged. First, the analysis was based on a single year of data (May 2024 to April 2025) due to application programming interface restrictions, which may not capture longer-term market cycles. Extending to multi-year datasets would enable stronger tests of robustness across regimes. Second, portfolio construction was limited to threshold-based selection criteria for risk and return metrics. Incorporating more sophisticated portfolio optimization techniques such as mean-variance optimization, could enhance adaptability. Third, this study did not include visual diagnostics, such as quantile fan charts or predictive price distribution plots, which could help illustrate asymmetries in forecast uncertainty. Incorporating these visual tools would enhance interpretability and allow a more detailed assessment of downside and upside risks in predictive modeling. Finally, the study focused solely on the Steam Market, which, while liquid, excludes third-party platforms with alternative pricing mechanisms and liquidity conditions. Studying these complementary markets would provide a more complete view of opportunities and risks in the CS2 trading ecosystem.

Future research could extend the analysis along several dimensions to strengthen the generalization and robustness of findings. First, expanding the time horizon beyond a single year would enable assessment of strategy performance across different market cycles. Second, future research should extend beyond the simple threshold-based portfolio construction used in this study. While this approach effectively demonstrated the feasibility and potential of AI-driven trading in the CS2 skin market, the application of more sophisticated portfolio optimization frameworks such as mean-variance optimization, Black-Litterman models, or robust optimization, could provide deeper insights into risk-return trade-offs and improve overall performance. Additionally, implementing a rolling-window training procedure would allow the model to incorporate new data over time, enhancing adaptability to market fluctuations and improving predictive robustness. Although this dynamic training setup was not applied here due to computational constraints and the exploratory focus on proof-of-concept validation, it represents a promising avenue for future work to further refine model performance and stability. Future research should extend this analysis to include more advanced architectures, such as Transformer-based models, convolutional neural networks, or foundation models like TimeGPT, to further enhance predictive performance and generalizability. Finally, extending the study beyond the Steam Market to include third-party exchanges with alternative pricing structures and liquidity conditions would provide a more comprehensive understanding of skin trading ecosystems.

## 5 Conclusion

This study successfully designed, developed, and validated an automated trading system tailored for CS2 skin trading. By applying financial performance metrics the system identifies and executes trades that align with predefined profitability and risk thresholds. First, through extensive backtesting across different portfolio sizes and time horizons, the analysis demonstrates AI consistently outperforms buy-and-hold in terms of ROI, and this advantage becomes stronger with longer horizons, although it is moderated by portfolio size. Second, diversification improves Sharpe Ratios for both approaches, yet AI portfolios systematically achieve higher risk-adjusted returns. Third, excess returns over buy-and-hold can be substantial in small portfolios and extended horizons, but the relative advantage decreases with greater diversification. These findings highlight the potential of AI-based trading strategies to generate superior profitability and risk-adjusted performance, particularly in targeted, concentrated portfolios rather than broad, highly diversified ones. By drawing parallels with equity markets, the results reinforce that even in alternative asset classes such as CS2 skins, the trade-offs between return, risk, and diversification conform to established principles of modern portfolio theory. AI strategies in CS2 trading behave much like mid-cap equity traders, systematically allocating toward moderately rare items that balance liquidity and predictive stability. By contrast, the buy-and-hold approach resembles a small-cap investor, accumulating rarer, less liquid skins whose long-term value is driven by scarcity premiums. Importantly, as portfolio size expands, diversification dampens rarity concentration, leading both strategies toward a composition that increasingly reflects the overall market distribution of skins.

## Data Availability

The data analyzed in this study is subject to the following licenses/restrictions: The datasets analyzed for this study can be found in the CSgoskins.gg. Requests to access these datasets should be directed to https://csgoskins.gg.
